# Sir James Y. Simpson

**Published:** 1870-06

**Authors:** 


***Sir James Y. Simpson,***

The celebrated Scotch author and practitioner, is dead. The profession and the world herein sustain an almost irremediable loss, which will be widely deplored. We have as yet no details. We suppose that in Boston his sudden demise will be attributed to his advocacy of Chloroform, and too little respect for the feelings of sundry of their magnates whom a recent letter of his disposed of summarily. It is to be hoped that the effort to falsify history by the Wells resolution, in the American Medical Association, may not have a similar disastrous effect upon any body.

